# Staplers versus energy devices for the intersegmental plane separation in thoracoscopic segmentectomy: a comparative study

**DOI:** 10.1186/s13019-022-02070-8

**Published:** 2022-12-17

**Authors:** Jiajun Han, Hongjie Yu, Haitao Ma

**Affiliations:** 1 Department of Thoracic Surgery, The First Affiliated Hospital of Soochow University, Suzhou, 215006 China; 2 Department of Thoracic Surgery, The Affiliated Suzhou Hospital of Nanjing Medical University, Suzhou Municipal Hospital, Suzhou, 215006 China; 3 Department of Thoracic Surgery, Suzhou Dushu Lake Hospital, Suzhou, 215006 China

**Keywords:** Lung segmentectomy, Energy device, Stapler, Intersegmental plane, Pulmonary function

## Abstract

**Background:**

In segmentectomy, in addition to the anatomy of the segmental hilum, the identification and separation of the intersegmental plane is also an important step of the operation. Because of its simplicity and high efficiency, most thoracic surgeons choose the staplers. But the energy devices also have its unique advantages in the separation of the intersegmental plane. This study compared the clinical efficacy of staplers and energy devices in the separation of the intersegmental planes during the uniport thoracoscopic segmentectomy through the clinical data.

**Methods:**

Clinical data of 89 patients undergoing uniport VATS lung segmentectomy from January 2019 to October 2020 at the First Affiliated Hospital of Soochow University were analyzed retrospectively. According to the different treatment methods of intersegmental plane, the patients were divided into two groups, 55 in the stapler group and 34 in the energy device group. The clinical data of the two groups were compared and analyzed statistically. And the univariate and multivariate logistic regression were also used to explore the influencing factors of postoperative complications.

**Results:**

Lung segmentectomy was successfully operated in both groups. There were statistically significant differences in operative duration, number of staplers used, surgical expenses and postoperative complications (*P* < 0.05). In terms of general data, including tumor location, operative hemorrhage, drainage volume on the first postoperative day, total postoperative drainage volume, postoperative chest tube retention duration, postoperative hospital stay, postoperative blood routine indexes, and postoperative pulmonary function indexes after 3 months, no significant differences were observed (*P* > 0.05). Smoking history (OR 5.08, 95% CI 1.05–24.56, *P* = 0.043) and intersegmental plane treatment (OR 3.18, 95% CI 1.11–9.14, *P* = 0.031) were risk factors for postoperative complications. Patients of the energy device group had a higher incidence of postoperative complications.

**Conclusions:**

In uniport thoracoscopic segmentectomy, the use of energy devices to treat the intersegmental plane will result in longer operative duration and higher postoperative complication rate, but it does not affect postoperative recovery and can help reduce surgical expenses. Both methods are safe and reliable. Clinically, the two methods can be reasonably selected according to the specific situation.

## Introduction

Lung cancer, as a disease with high morbidity and mortality, has become the leading cause of cancer death [1] and seriously threatens people's lives and health. With the continuous updating of medical imaging equipment and the global spread of COVID-19, the popularizing rate of CT is getting higher and higher, and more patients with early lung cancer have been found. With the result of JCOG-0802 research, segmentectomy should be the standard surgical procedure for the patients with small-sized peripheral NSCLC [2]. In addition to the technical difficulties of the anatomy of the segmental hilum and the identification of the intersegmental plane, the proper treatment of the intersegmental plane is also a focus of discussion. The stapler is widely used because of its high efficiency and convenience. But the energy device has also shown its advantage in flexibility with the advancement and innovation of technology.

In this study, by means of three-dimensional imaging technology and retrospective analysis of relevant data, the clinical efficacies of staplers and energy devices in the separation of intersegmental planes during uniport thoracoscopic segmentectomy are analyzed and compared, so as to guide clinical application.

## Methods

### Study design and patient characteristics

The study was conducted in accordance with the Declaration of Helsinki (as revised in 2013). This study was approved by the Ethics Committee of the First Affiliated Hospital of Soochow University (Approval No. 2022 technology 195) and informed consent was taken from all the patients.

The clinical data of 89 patients undergoing uniportal VATS lung segmentectomy from January 2019 to October 2020 at the First Affiliated Hospital of Soochow University were analyzed. Preoperative examinations were performed to exclude surgical contraindications. There were 55 patients in the stapler group, for whom the staplers of Johnson & Johnson were used to separate the intersegmental plane during the operation. And other 34 patients were in the energy device group, for whom only electrotome and ultrasonic scalpel were used. All operations were performed by the same team of surgeons.

#### The inclusion criteria

(1) The lesion was < 2 cm in diameter and located in the center of the target lung tissue. Wedge resection was unfeasible. Lung segmentectomy could ensure adequate surgical margin. (2) Lobectomy was contraindicated under the circumstances that the lung function was poor or there were other serious diseases; (3) At least one of the following three characteristics should be met, which were, the postoperative pathology was adenocarcinoma in situ; CT showed that the ground glass composition of nodules was ≥ 50%; Nodules doubling time ≥ 400 d. (4) No obvious surgical contraindications were found in preoperative examination.

#### Exclusion criteria

(1) Patients in poor physical conditions who were unable to tolerate the operation. (2) Past history of pulmonary surgery. (3) Prolonged operative duration due to intraoperative thoracic dense adhesions.

### Interventions

#### Operations

All patients underwent uniportal VATS lung segmentectomy. 3D reconstruction was performed preoperatively based on the patient’s high-resolution CT. The patient was placed on the unaffected side under general anesthesia. The double-lumen endotracheal tubes were intubated. The unaffected-side lung was ventilated. The utility incision was usually made at the fourth intercostal space, anterior axillary line of the upper lung; as for the lower lung, it was usually made at the fifth intercostal space, anterior axillary line. During the surgery, the pulmonary segmental arteries, veins and segmental bronchus were precisely identified according to the preoperative 3D reconstruction, and were severed respectively. The artery was ligated at the proximal end with a silk thread and then severed by an ultrasonic scalpel or a white stapler, the vein was ligated at the proximal end with a silk thread and then severed by an ultrasonic scalpel or a white stapler, and the segmental bronchus was severed by a blue stapler. The intersegmental plane was identified by the modified inflation-deflation of the lung method, and marked on the lung surface with an electrocoagulation hook.

**Fig. 1:**
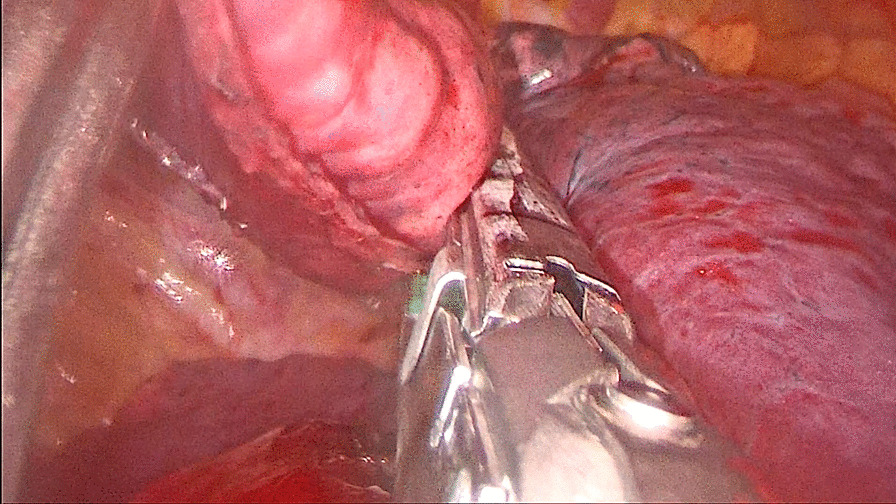
The staplers were used to separate the targeted lung segment

**Fig. 2:**
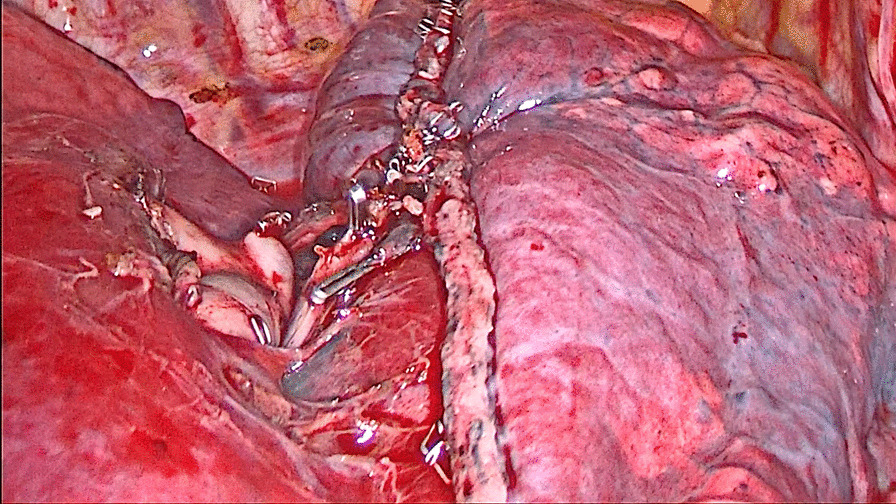
The intersegment plane separated by the staplers

**Fig. 3:**
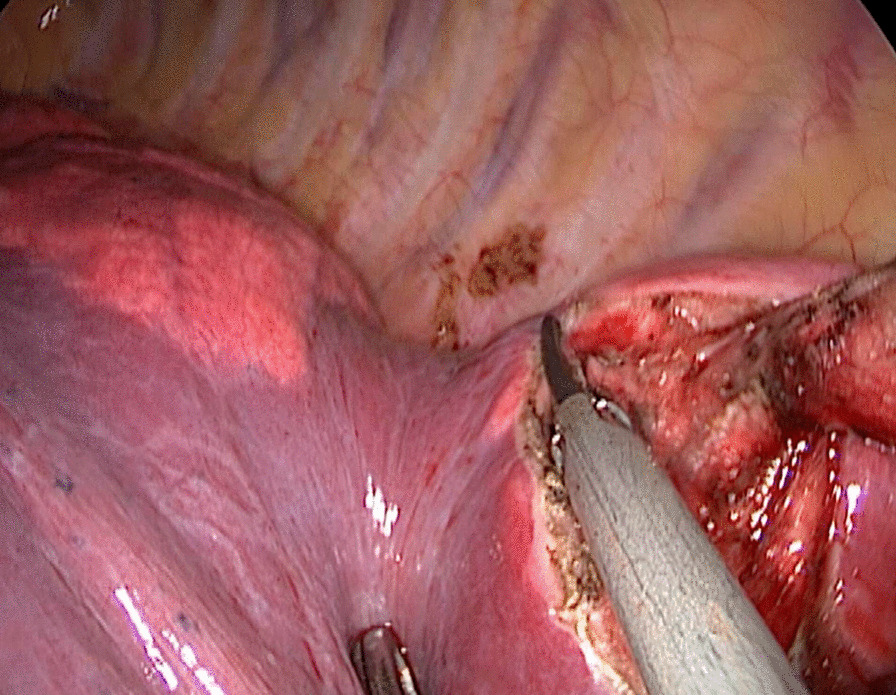
The ultrasonic scalpel was used to separate the targeted lung segment

**Fig. 4:**
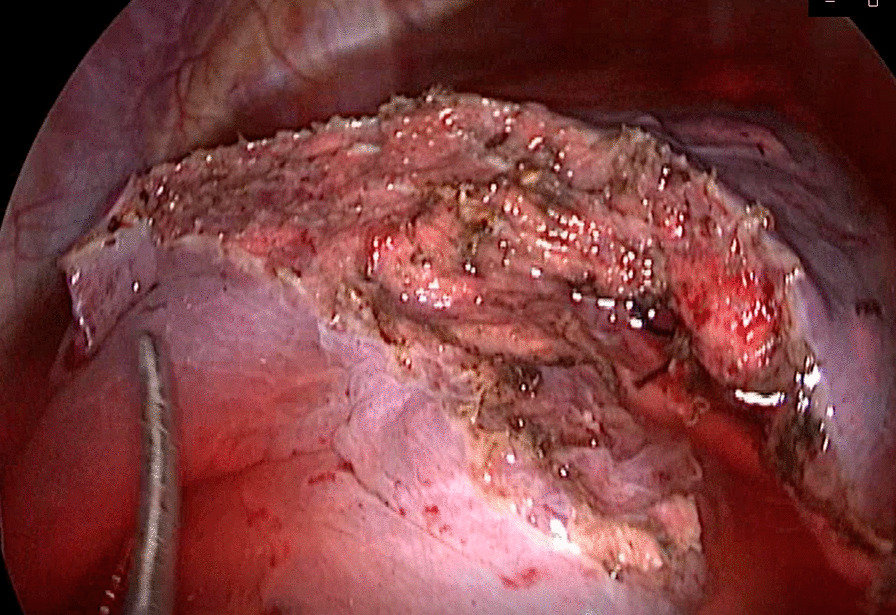
The intersegment plane separated by the ultrasonic scalpel and the electrocoagulation hook

For patients in the stapler group, the staplers were used to separate the targeted lung segment, which was showed in Figs. 1 and 2, while for patients in the energy device group, the ultrasonic scalpel and the electrocoagulation hook were used, which was showed in Figs. 3 and 4.

The specimen was removed. It was sent for the fast freezing pathological examination after the focus of infection had been marked and the margins had been confirmed to meet the requirements. While waiting for the pathological results, points with obvious air leakage would be sutured. The lymph nodes were sampled in cases of adenocarcinoma in situ carcinoma, microinvasive adenocarcinoma or invasive adenocarcinoma. One chest tube was placed after surgery.

### Assessments

The basic information and perioperative data were collected, including age, gender, smoking history, tumor location, tumor size, postoperative pathology, operative duration, operative hemorrhage, number of staplers used, drainage volume on the first postoperative day, total postoperative drainage volume, postoperative chest tube retention duration, postoperative hospital stay, postoperative complications, surgical expenses, postoperative blood routine indexes, and postoperative pulmonary function indexes after 3 months.

The indications for extubation were no pneumothorax or atelectasis, no air leakage, water fluctuating little in the chest tube, pleural effusion is not dark in color, and a daily drainage volume of ≤ 200 ml.

The postoperative complications mainly included atelectasis, pulmonary infection and air leakage. Air leakage levels were applied as follows: Level 0 = never, Level 1 = coughing, Level 2 = deep breath, and Level 3 = always.

### Statistical analysis

Statistical analyses were performed with IBM SPSS 19.0 software package (IBM, Armonk, NY, USA). For quantitative data, the distribution morphology and homogeneity of variance were verified. If the data were normally distributed, the Student’s t-test was compared and represented by the mean ± standard deviation (mean ± SD); otherwise, median (interquartile spacing) [M (IQR)], the Mann–Whitney U test was applied. Categorical data was compared using the chi-square (χ2) test or Wilcoxon rank-sum test. Association data were analyzed using odds ratio (OR) and 95% confidence interval (CI) analysis. The logistic regression model of stepwise regression was used for univariate and multivariate analysis, and variables with *P* < 0.2 in univariate analysis were included in multivariate analysis. *P* -value < 0.05 was considered statistically significant.

## Results

The operations of all the 89 patients were completed successfully. The location of the segmentectomy was showed in Table 1. There were no significant differences statistically in general data between the two groups (*P* > 0.05, Table 2).

**Table 1:** Location of the segmentectomy

Segmentectomy	Stapler group	Energy device group
Right lungs		
S1	10	3
S2	4	2
S3	4	3
S1 + 2	0	1
S4	0	3
S6	1	2
S8	2	0
S9	0	1
S10	0	1
S9 + 10	0	2
Left lungs		
S1	1	0
S1 + 2	7	4
S3	5	0
S4 + 5	4	1
S1 + 2 + 3	3	0
S6	14	4
S8	0	3
S9	0	1
S10	0	2
S9 + 10	0	1

**Table 2:** Characteristics and difference of the patients

Characteristics	Stapler group	Energy device group	*P*-value
Age (years)	51.25 ± 12.81	50.29 ± 12.66	0.723
Gender			0.657
Male	22	12	–
Female	33	22	–
Height (cm)	164 ± 8.70	165.26 ± 8.81	0.509
Weight (kg)	62.07 ± 10.07	62.09 ± 10.43	0.994
Smoking history	8	4	0.709
Basic diseases^a^	11	12	0.109
Hypertension	7	5	–
Diabetes	4	6	–
Malignancy	2	3	–
Bronchiectasis	1	0	–
COPD	1	0	–
Preoperative albumin	43.64 ± 4.85	42.27 ± 4.25	0.177
Tumor location			0.017^b^
RUL	18	9	–
RML	0	3	
RLL	3	6	–
LUL	20	5	–
LLL	14	11	–
Difficulty of segmentectomy^c^			0.058
Easy	22	7	–
Difficult	33	27	–
Tumor size (mm)	10 (6)	8 (3)	0.058
Pathological diagnose			0.092
Benign	12	3	–
AIS	3	3	–
MIA	26	24	–
IAC	14	4	–

*COPD* chronic obstructive pulmonary disease; *RUL* right upper lobe; *RML* right middle lobe; *RLL* right lower lobe; *LUL* left upper lobe; *LLL* left lower lobe; *AIS* adenocarcinoma in situ; *MIA* minimally invasive adenocarcinoma; *IAC* invasive adenocarcinoma

^a^Basic diseases include hypertension, diabetes, malignancy, bronchiectasis, and COPD

^b^Correction *P* = 0.005

^c^Easy segmentectomy is defined as resection of only one intersegmental plane, including LS1 + 2 + 3, LS4 + 5, LS6, LS8 + 9 + 10, RS6, and RS7 + 8 + 9 + 10. Rest of them is defined as difficult segmentectomy

During the perioperative period, there were no significant differences in operative hemorrhage, drainage volume on the first postoperative day, total postoperative drainage volume, postoperative chest tube retention duration, and postoperative hospital stay between the two groups (*P* > 0.05). In the stapler group, the mean operative duration was (147.84 ± 35.34) min, the number of staplers used was 8 (2), and the mean surgical expenses were 32,320 (6460) CNY. In the energy device group, the mean operative duration was (210.53 ± 74.56) min, the number of staplers used was 2 (2), and the mean surgical expenses were 12,940 (6460) CNY. The three items of data were statistically different (*P* < 0.05, Table 3).

**Table 3:** Perioperative related factors in all the patients

Factors	Stapler group	Energy device group	*P*-value
Mean operative duration (minutes)	147.84 ± 35.34	210.53 ± 74.56	< 0.001
Mean operative hemorrhage (ml)	20 (10)	20 (0)	0.070
Mean drainage volume on the first postoperative day (ml)	75 (75)	80 (50)	0.888
Total postoperative drainage volume (ml)	527 (500)	577.5 (266)	0.265
Postoperative chest tube retention duration (days)	5 (2)	5 (2)	0.224
Postoperative hospital stay (days)	5 (3)	5 (3)	0.118
Number of lymph nodes	5 (3)	6 (5)	0.230
Number of staplers used	8 (2)	2 (2)	< 0.001
Surgical expenses (CNY)	32,320 (6460)	12,940 (6460)	< 0.001

There were no significant differences in inflammatory indexes like white blood cells, neutrophils, neutrophil percentage and NLR between the two groups, which were tested before and after surgery (*P* > 0.05, Table 4). There were also no significant differences in pulmonary function indexes like FEV1, FEV1%, the ratio and difference of FEV1 and FEV1% between the two groups which were tested before and after surgery (*P* > 0.05, Table 5).

**Table 4:** Inflammatory indexes in all the patients

Factors	Stapler group	Energy device group	*P*-value
Before Surgery			
White blood cells (10^9/L)	5.47 ± 1.33	5.10 ± 1.26	0.196
Neutrophils (10^9/L)	3.21 ± 1.03	2.97 ± 1.10	0.304
Neutrophil percentage (%)	58.0 ± 7.3	57.0 ± 7.6	0.531
NLR	1.75 (0.92)	1.64 (0.60)	0.269
After Surgery			
White blood cells (10^9/L)	10.22 (3.50)	9.29 (2.78)	0.282
Neutrophils (10^9/L)	8.24 (3.49)	7.64 (2.94)	0.384
Neutrophil percentage (%)	80.5 ± 6.4	80.5 ± 6.2	0.985
NLR	6.60 (3.58)	5.73 (5.28)	0.813

*NLR* neutrophil to lymphocyte ratio

**Table 5:** Pulmonary function indexes in all the patients

Factors	Stapler group	Energy device group	*P*-value
FEV1 (L)			
Before surgery (A) (L)	2.77 ± 0.78	2.84 ± 0.79	0.678
After surgery (B) (L)	2.37 ± 0.62	2.46 ± 0.77	0.542
B/A	0.86 ± 0.04	0.87 ± 0.05	0.532
B-A (L)	0.37 ± 0.12	0.35 ± 0.12	0.455
FEV1%			
Before surgery (C)	98.2 ± 15.3	99.0 ± 13.8	0.798
After surgery (D)	85.9 ± 13.0	86.0 ± 13.3	0.976
D-C	13.2 ± 4.7	12.5 ± 4.7	0.525

FEV1: forced expiratory volume in one second

The complications occurred in 14 (25.5%) cases in the stapler group, which 12 (21.8%) of them were air leakage, and in 16 (47.1%) cases in the energy device group, which all of them were air leakage. The two items of data were statistically different (*P* < 0.05). But most of the air leakage were I degree, and this was the only data of air leakage degree which was statistically different (*P* < 0.05). Specific values are shown in Table 6.

**Table 6:** Complications in all the patients

Factors	Stapler group	Energy device group	*P*-value
Complications	14 (25.5%)	16 (47.1%)	0.036
Atelectasis	1	0	0.429
Pulmonary infection	1	0	0.429
Air leakage	12	16	0.013
I degree	11	16	0.007
II degree	1	0	0.429
III degree	0	0	-
Air leakage duration	1 (0)	1 (1)	0.501

Univariate and multivariate logistic regression was performed on age, gender, height, weight, smoking history, basic diseases, tumor size, plane treatment and FEV1% (before surgery). The results of univariate analysis showed that plane treatment was the influencing factor of postoperative complications (*P* < 0.05). The variables with *P* < 0.2 in univariate regression were further included in multivariate logistic regression, the results showed: smoking history (OR 5.08, 95% CI 1.05–24.56, *P* = 0.043) and plane treatment (OR 3.18, 95% CI 1.11–9.14, *P* = 0.031) were risk factors for postoperative complications, and a history of smoking or the use of energy devices to separate the intersegmental plane had a higher incidence of postoperative complications. The results are shown in Table 7.

**Table 7:** Univariate and multivariate logistic regression of postoperative complications

Factors	OR(95%CI)	*P*-value
*Univariate Logistic Regression*		
Age (years)	1.02 (0.98–1.06)	0.285
Gender	0.91 (0.37–2.25)	0.832
Height (cm)	1.00 (0.95–1.05)	0.887
Weight (kg)	1.01 (0.97–1.05)	0.692
Smoking history	3.29 (0.94–11.44)	0.061
Basic diseases	0.456 (0.15–1.38)	0.165
Tumor size (mm)	1.11 (0.98–1.27)	0.106
Plane treatment, energy devices	2.60 (1.05–6.44)	0.039
FEV1% (before surgery)	1.02 (0.99–1.05)	0.194
*Multivariate Logistic Regression*		
Smoking history	5.08 (1.05–24.56)	0.043
Basic diseases	0.28 (0.08–1.04)	0.057
Tumor size (mm)	1.14 (0.98–1.33)	0.091
Plane treatment, energy devices	3.18 (1.11–9.14)	0.031
FEV1% (before surgery)	1.02 (0.98–1.06)	0.257

## Discussion

With the extensive application of CT and the wide spread of the concept of early diagnosis and treatment of lung cancer, a large number of early-stage lung cancer patients have been found [3]. Emerging treatments such as radiofrequency ablation and stereotactic radiotherapy have brought hope to a large number of patients with deep and multiple nodules. Nevertheless, surgery is still the main treatment for early-stage lung tumors. For deep lesions, wedge resection sometimes fails to achieve surgical objectives, and lobectomy will bring excessive surgical traumas. Therefore, segmentectomy is now increasingly used by thoracic surgeons. Combined with related researches [4, 5], the same therapeutic effect can be achieved on patients with early lung disease. Resection of fewer lung tissues means the preservation of more lung functions, which will improve patients' postoperative life quality [6]. Due to the lack of natural anatomical boundaries on lung segments, it is difficult to identify of intersegmental plane. And the identified intersegmental plane should be segmented in a suitable way. At present, the staplers and the energy devices are two mainstream tools for segmentation. Both options have their advantages and disadvantages.

The advantage of using the staplers to separate the intersegmental plane lies in its convenience and quickness. The operative duration can be saved, as well as the anesthesia duration. A reliable stapler can help reduce the risk of bleeding and air leakage at the intersegmental plane, but the strong compression from the stapler entails the risk of recruitment difficulties for lung tissues near the cutting edge [7]. And the presently used staplers have limitation in angles. This problem is particularly significant when the uniport thoracoscopic surgery is being performed. A perfect fit with the marked intersegment plane cannot be achieved during the separation. This may result in insufficient incision margins due to a too small incision, and accidental damage to the intersegmental veins and the lung tissues in other lung segments due to an excessive incision. The polygonal shape of intersegmental plane also makes it impossible to use excessive staplers regardless of price.

On the other hand, the energy device, because of its flexibility in angles and small processing surface, can better fit the actual intersegmental plane, and the remaing lungs will have better recruitment and plasticity [8]. The combination usage of electrotome and ultrasonic scalpel can ensure a certain separation speed and achieve better hemostasis effect at the same time. However, due to the limited area of single operation, the total operative duration will be definitely increased. In terms of price comparison between the staplers and the energy devices, the total price of the staplers required during the operation is bound to exceed that of the electrotome and ultrasonic scalpel.

In this study, it is found by comparison analysis that, although the usage of energy device will increase the operative duration and the probability of I degree air leakage after operation, the number of staplers required will be decreased and the surgical expenses will be reduced. The energy device group requires longer operative duration for the separation of the intersegment planes and the reinforced suture at the obvious air leakage points, and eschars and thermal damage will be occurred on the intersegment planes [9, 10]. There is no significant difference in inflammatory indexes and the probability of postoperative infection between the two groups, which indicates that energy devices will not affect the postoperative recovery of patients.

The incidence of postoperative air leakage in the energy device group is higher, and the multivariate logistic analysis further confirms that the energy device is a risk factor for postoperative complications, but most patients have I degree air leakage, which does not last for a long time (the median number of days is 1 day) and the postoperative hospital stay will not be increased, so there is no significant statistical difference between the two groups.

In the comparison of patients’ lung function three months after surgery, there is no significant statistical difference between the two groups, which does not reflect the advantages of energy devices. This result is consistent with the findings of Ohtsuka T and Miyasaka Y [11]. The result may be related to the following situations: 1. The eschars caused by the electrotome and the lung tissue damage caused by the heat transfer of the ultrasonic scalpel, affect the recruitment and functional recovery of the lung. 2. The loss of segmental lung function is little, and the proportion of each segment is different, and due to the small sample size, it is difficult to make an accurate comparative study. But according to the current results, both methods are safe and reliable.

In clinical practice, it is necessary to choose the appropriate treatment according to the patients’ individual situation. For the elderly patients, or those with underlying lung diseases, the anesthesia and operative duration should be reduced as much as possible to lower the risk of postoperative air leakage. Therefore the staplers could be the first choice for them. For patients with good general condition and surgical tolerance, but with poor economic condition, the energy devices can be chosen. For complex lung segments, such as there are multiple segment surfaces at the basal segment of the lung and the segmental hilum is not clearly exposed, the use of energy devices can effectively reduce the occurrence of accidental injury. Chen et al. reported the combined method of staplers and energy devices. The energy devices can be used in the thicker segmental tissues, while the staplers are suitable for the distal lung tissues [12]. More clinical cases are required to verify the efficacy of the combination of the two methods.

## Conclusions

In conclusion, it is safe and reliable to use the staplers or the energy devices to segment the intersegment plane, and the tools should be reasonably selected according to the patient's own situation.

## Data Availability

The datasets used and/or analysed during the current study are available from the corresponding author on reasonable request.

## Abbreviations

VATS: Video assisted thoracoscopic surgery
COVID-19: Corona virus disease 2019
CT: Computed tomography
3D: Three dimensional
